# Moderating influences of baseline activity levels in school physical activity programming for children: the Ready for Recess project

**DOI:** 10.1186/1471-2458-14-103

**Published:** 2014-02-01

**Authors:** Pedro F Saint-Maurice, Gregory J Welk, Daniel W Russell, Jennifer Huberty

**Affiliations:** 1 Iowa State University, Department of Kinesiology, Ames, IA 50011, USA; 2 Iowa State University, Department of Human Development and Family Studies, Ames, IA 50011, USA; 3 School of Nutrition & Health Promotion, Arizona State University, Phoenix, AZ 85004-0698, USA

**Keywords:** PA promotion, MVPA, Recess, Youth

## Abstract

**Background:**

A limitation of traditional outcome studies from behavioral interventions is the lack of attention given to evaluating the influence of moderating variables. This study examined possible moderation effect of baseline activity levels on physical activity change as a result of the Ready for Recess intervention.

**Methods:**

Ready for Recess (August 2009-September 2010) was a controlled trial with twelve schools randomly assigned to one of four conditions: control group, staff supervision, equipment availability, and the combination of staff supervision and equipment availability. A total of 393 children (181 boys and 212 girls) from grades 3 through 6 (8–11 years old) were asked to wear an Actigraph monitor during school time on 4–5 days of the week. Assessments were conducted at baseline (before intervention) and post intervention (after intervention).

**Results:**

Initial MVPA moderated the effect of Staff supervision (β = −0.47%; p < .05), but not Equipment alone and Staff + Equipment (p > .05). Participants in the Staff condition that were 1 standard deviation (SD) below the mean for baseline MVPA (classified as “low active”) had lower MVPA levels at post-intervention when compared with their low active peers in the control condition (Mean _diff_ = −10.8 ± 2.9%; p = .005). High active individuals (+1SD above the mean) in the Equipment treatment also had lower MVPA values at post-intervention when compared with their highly active peers in the control group (Mean _diff_ = −9.5 ± 2.9%; p = .009).

**Conclusions:**

These results indicate that changes in MVPA levels at post-intervention were reduced in highly active participants when recess staff supervision was provided. In this study, initial MVPA moderated the effect of Staff supervision on children’s MVPA after 6 months of intervention. Staff training should include how to work with inactive youth but also how to assure that active children remain active.

## Background

There is considerable public health interest in developing and testing strategies to help youth be more physically active [1]. School-based interventions are a common target due to the opportunity to reach large numbers of youth and the availability of staff and resources [2], but unfortunately, the success of school-based programming is limited [1,3].

A limitation of traditional analyses of school-based interventions is that the focus is on evaluating only the overall group-level effects. This provides a limited perspective of the outcomes since it is possible for interventions to have differential effects within the sample population. An intervention, for example, might have benefits for inactive youth but not for others. It is therefore important to conduct follow-up evaluations of the data collected to examine possible moderating influences.

Members of our team recently reported on the outcomes from a recess-based intervention, the Ready-for-Recess (R4R) project, a controlled trial evaluating environmental modifications to promote physical activity during recess [4]. Recess plays a critical role in children’s development and well being [5,6] and recess time has also been shown to make important contributions to children’s school and total daily activity [7-10]. Ridgers et al., 2012, recently conducted a systematic review to examine activity levels during recess. The review emphasized the need to explore different activity promotion strategies and how these can impact the behaviors of important subgroups (e.g. boys vs. girls) [11]. Other recess-based studies have examined the benefits of adult supervision [12], and equipment availability during recess [13], but a unique aspect of R4R was that it evaluated the independent and combined effects of adult supervision and equipment on physical activity during recess. Schools in the R4R study (n = 12) were randomly assigned to one of four conditions: control group, staff supervision, provision of equipment, and the combination of staff supervision and equipment. The combination of staff supervision and equipment availability led to a significant increase of moderate-to-vigorous physical activity (MVPA) in boys (+14.1%) [4]. These findings suggest that recess interventions can be successful when promoting active behaviors among groups of children.

As with most clinical trials [14], the success of R4R was examined using group-level comparisons with the rate of change across time being defined as a fixed effect. This approach assumes that the amount of change across individuals is the same. While this fixed effects approach is appropriate for the overall evaluation, further work is required so that the assumption of homogeneity across individuals can be examined and to avoid what is defined as the “*ecological fallacy*” [15,16].

The inter-individual variability in the context of a PA intervention can be examined by allowing the impact of the interventions to be defined as random in the regression model. A test of homogeneity is used to determine if the rate of change across time is consistent across individuals. If there is variability between individuals one can try to explain this variability and thereby better understand individual determinants of PA. This approach can be undertaken using latent growth curve analysis, which is similar to the classic fixed effects model but with the advantage that researchers can specify predictors in the model as random factors [16]. This approach makes it possible to systematically evaluate factors influencing the consistency of change across individuals.

The purpose of this study was to examine if baseline levels of moderate-to-vigorous physical activity (MVPA) moderated the effects from the various R4R treatments.

## Methods

### Intervention design

The R4R (August 2009-September 2010) project evaluated the independent and combined effects of staff training and equipment on children’s participation in MVPA during daily recess. Twelve schools were randomly assigned to one of four conditions: Staff Training (ST), Equipment (EQ), Staff Training + Equipment (STEQ) or a control condition. We have published a main outcome paper that provides additional details about the R4R intervention [4].

### Participants

Elementary children (3rd-6th grades, 8–11 years of age) from 12 participating schools were recruited to participate in the evaluation of the R4R intervention. Demographic information obtained from the school included: age, gender, birth date, race, and free and reduced lunch status. The Ready for Recess intervention study was approved by the Institutional Review Board at University of Nebraska at Omaha. Children that returned signed consent forms were enrolled in the study.

### Anthropometric measures

Height and weight data on participants were obtained by the research team using standard field based techniques. However, we had to minimize the disruption of the school schedule at some of the schools and therefore were not able collect this information on the entire sample involved in the study.

### Physical activity - accelerometer measure

Physical Activity was assessed with the GT1M Actigraph Accelerometers (Actigraph, Pensacola, FL). Students were asked to wear the activity monitors during the school day (approximately 8:00 AM – 3:00 PM), for 4–5 consecutive days. Members of the research team put the monitors on the children each morning and took them off at the end of the school day to ensure compliance. Teachers provided logs of student attendance and a daily schedule to record time periods for recess, lunch, and physical education.

The data from the Actigraphs were recorded in 5 second epochs and processed using Freedson et al. (2005) age-specific cutpoints (METs = 2.757 + (0.0015 · counts · min-1) – (0.08957 · age [yr]) – (0.000038 · counts · min-1 · age [yr]) [17]. Each epoch during recess was classified as light (1.5 - 3.9 METs), moderate (MPA; 4.0 - 6.0 METs) or vigorous (VPA; > 6.0 METs) and the data were then aggregated to determine time spent in LPA, MPA, VPA and MVPA (MPA + VPA). Participants were included in the final analyses if they were at school (and had data) on at least 3 of the 5 days. Since the activity monitors were only used during school time it was assumed that students wore the monitors during all time unless indicated otherwise by school teachers. Activity outcomes were computed as an average of the valid data extracted from recess periods. The major computed outcome measure was the percent time allocated to MVPA in order to standardize for different lengths of recess.

### Data analysis

Latent Growth Curve (LGC) models were used to account for the longitudinal nature of the data (repeated-measures) and potential individual differences (nested within schools) when exposed to the different treatments. The variable Trial (coded as 0 = trial 1, 1 = trial 2) was defined as a level-1 predictor (growth factor), characteristics of the children as level-2 predictors, and schools as level-3 predictors. Post-intervention average percent time in MVPA during recess (activity obtained from valid recess segments) was defined as the main outcome of interest (dependent variable). Level-2 covariates included grade (coded with 0 = 3rd grade, 1 = 4th grade, 2 = 5th grade, and 3 = 6th grade), gender (coded 0 = male and 1 = female), average recess duration (in minutes), and baseline (trial 1) percent time in MVPA during recess. The three treatments were used as level-3 predictors and coded using dummy coding (variable Staff coded 0 = No Staff, 1 = Staff; variable Equipment coded 0 = No equipment, 1 = Equipment; and variable Staff + Equipment coded 0 = No Staff + Equipment, and 1 = Staff + Equipment). Other demographic characteristics of the children such as BMI, race, or social economic status were not included since this information was not available for the entire sample. Only individuals with complete activity data at each assessment (Trial) were included in the analysis.

To examine the direction, magnitude and consistency of the moderation of baseline MVPA on each intervention, least-square means comparing each treatment to the control group were computed (using the final LGC model) at three fixed values based on baseline MVPA scores: Mean - 1 standard deviation, Mean, and Mean + 1 standard deviation (see magnitude and direction of moderation effect). These three categories were operationalized as Low Active, Moderate Active, and High Active, respectively. These comparisons were performed to examine the overall trend over time (across treatments and control group) and also separately for each activity group. A total of 12 least-square means tests with their respective mean difference (Mean _diff_), standard errors and p-values are reported.

Results from the nested models (test of model fit for level-1 and level-2 predictors) and moderation effects were computed with 95% confidence intervals (p < .05). To account for the increased type I error associated with multiple statistical tests when comparing subgroups of activity, statistical analysis associated with least-square means were performed with a 99% confidence interval and therefore defined as significant if p-value < .01. In addition, mean differences for these analyses were considered borderline significant if p-value was between .01 and .05. Statistical procedures as described above were performed using SAS v9.2 (Cary, North Carolina).

## Results

From the total sample of 667 participants, there were 393 participants (181 boys and 212 girls) who had three days of activity monitor data on both trials. The sample size differs from the main outcome results previously reported by Huberty et al., 2013, since it was important to have robust estimates of individual variability patterns across time. The distribution of participants across gender, grade and intervention is provided in Table 1.

**Table 1:** Distribution (N) of gender and grade per intervention group

	**Gender**			**Grade**			
	**Boys**	**Girls**	**3rd**	**4th**	**5th**	**6th**	**Total**
Control	47	45	20	27	24	21	**92**
ST	39	69	32	19	34	23	**108**
EQ	59	51	23	27	38	22	**110**
STEQ	36	47	8	22	23	30	**83**
**Total**	**181**	**212**	**83**	**95**	**119**	**96**	**393**

ST = Staff treatment; EQ = Equipment treatment; STEQ = Staff + Equipment treatment.

### Inter-individual variability and test of covariates

**Figure 1:**
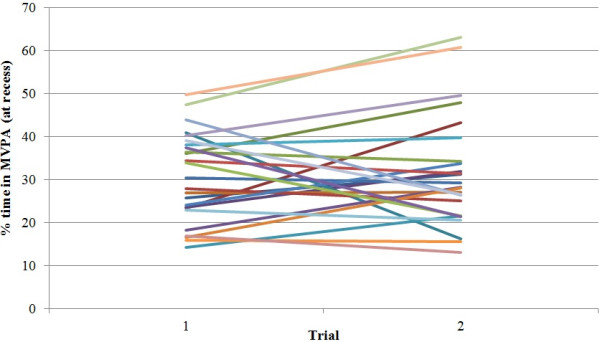
**Percent time spent in MVPA at recess for a (randomly selected) subgroup of 25 individuals.** Each line represents an individual. This figure illustrates a high degree of variability in individual’s baseline scores (intercept) and change over time (slopes). This justifies the need for a random intercept term at level-2 (individual).

**Figure 2:**
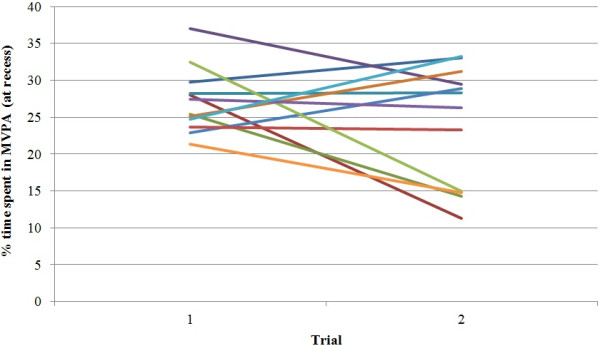
**Percent time spent in MVPA at recess for the 12 schools.** Each line represents a school. Similarly to Figure 1, there was a significant difference in baseline scores for school clusters, and therefore, justifying the need for random intercepts at this level.

Preliminary analyses were conducted to test assumptions needed for the use of LGC. We examined the variability in the MVPA scores across time to ensure that there was sufficient variability in the data. The intraclass correlation for individuals was equal to .08, while for schools it was equal to .07 and the covariance parameters indicated that intercept scores for time spent in MVPA were significantly different between individuals (see Figure 1) and schools (see Figure 2) (p < .05). Additional testing with level-1 and level-2 predictors revealed significant improvements in the fit of the model (indicated by a significant (p < .05) change in the (LLR) value). The results supported the clustered nature of the data and differences on baseline activity levels between individuals and schools. These findings support the planned model used to examine the moderator effect of baseline activity on change in MVPA levels associated with each condition.

### The latent growth curve model

Interpretation of the latent growth curve model b-weights (Table 2) revealed that change in MVPA from trial 1 to trial 2 in the control group (holding all the other predictors in the model constant) could be explained by initial MVPA scores. Per each unit increase in baseline MVPA score, there was a reduction of 0.76% on MVPA scores change from trial 1 to trial 2 (e.g., children in the control group with lower baseline MVPA scores had greater positive changes in MVPA from trial 1 to trial 2 while the opposite was true for children with higher baseline MVPA scores). Similar trial X baseline MVPA interactions with each condition revealed that this trend was attenuated by ST (β = 0.29 ± 0.12, p = .02) but not EQ (p = .35) or STEQ (p = .74). In other words, youth in the Staff condition that were less active at baseline had greater MVPA differences (from their respective control group) in MVPA at trial 2 when compared to their more active peers. Per each unit increase in percent MVPA at baseline, there was an attenuation of the ST effect by 0.47% in MVPA levels at trial 2.

**Table 2:** Regression coefficients for the final latent growth curve model

	**Estimate**	**SE**	**df**	**t-value**	**p-value**
**Intercept**	−6.73	5.62	8	−1.20	0.27
**b-weights**					
Trial	20.35	3.00	759	6.78	<.001
Baseline MVPA	1.72	0.15	759	11.72	<.001
Gender	−2.39	0.73	759	−3.26	0.001
Grade	−2.32	0.35	759	−6.69	<.001
Recess duration	0.06	0.07	759	0.84	0.4
ST	17.90	6.71	8	2.67	0.03
EQ	8.41	6.60	8	1.27	0.24
STEQ	−3.07	7.36	8	−0.42	0.69
Trial X ST	−15.73	3.96	759	−3.97	<.001
Trial X EQ	−6.49	3.90	759	−1.66	0.1
Trial X STEQ	5.39	4.39	759	1.23	0.22
Trail X baseline MVPA	−0.76	0.09	759	−8.32	<.001
ST X baseline MVPA	−0.36	0.2	759	−1.85	0.06
EQ X baseline MVPA	0.11	0.20	759	0.57	0.57
(STEQ) X baseline MVPA	0.01	0.20	759	0.05	0.96
Trial X ST X baseline MVPA	0.29	0.12	759	2.39	0.02
Trial X EQ X baseline MVPA	−0.12	0.12	759	−0.94	0.35
Trial X (STEQ) X baseline MVPA	−0.05	0.14	759	−0.34	0.74

SE = standard error; df = degrees of freedom.

ST = Staff treatment; EQ = Equipment treatment; STEQ = Staff + Equipment treatment.

### Magnitude and direction of the moderation effect

The overall mean score (across treatments and control group) for percent of time spent in MVPA at baseline was 27.5%. Predicted values were estimated by fixing baseline activity values in the final model as 27.5%, 12.5% (−1SD), and 42.4% (+1SD). These predicted activity scores represented Moderate, Low, and High Active groups, respectively. There were significant changes over time for the three groups but the patterns were very different. The Low Active group had a significant increase in MVPA from trial 1 to trial 2 (Mean _diff_ = 6.3 ± 0.9%; t (11) = 6.7; p < .001), while the Moderate (Mean _diff_ = −4.5 ± 0.7%; t (11) = −6.8; p < .001) and High active group (Mean _diff_ = −15.3 ± 0.9%; t (11) = −16.2; p < .001) decreased their MVPA levels (p < .001).

**Figure 3:**
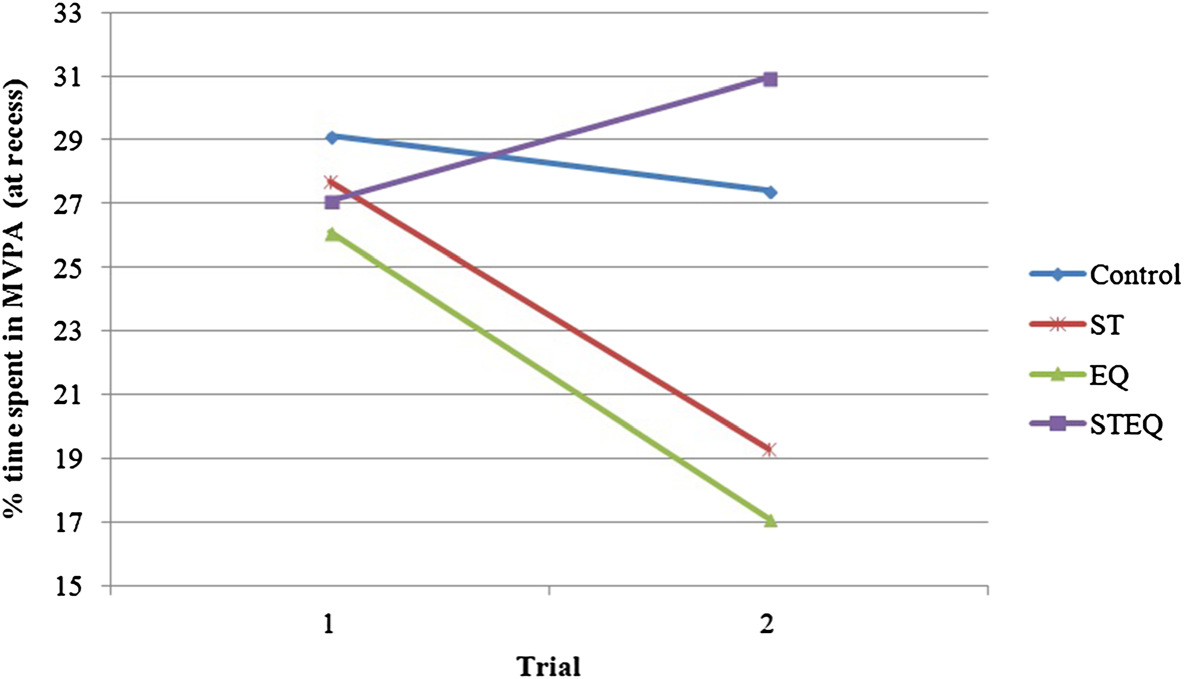
**Average MVPA scores for the control and each treatment group.** Both the Staff (ST) and the Equipment (EQ) treatments had a negative effect on individuals’ MVPA. The Staff + Equipment (STEQ) treatment effect was positive.

Further evaluation, however, shows that the response varies depending on the treatment group. When stratified by treatment group, the mean baseline MVPA values (Medium) for the ST, EQ and STEQ treatments were 27.7%, 26.1%, and 27.1%, respectively. The corresponding values for Low Active (−1SD) were equal to, 12.2%, 11.0%, and 13.3% while values for High Active (+1SD) were 43.2%, 41.3%, and 40.8%. The values for Low Active were all similar at baseline (Trial 1) but the levels of MVPA at trial 2 in the ST schools were significantly lower than the respective Low Active control group (Mean _diff_ = −10.8 ± 2.9%; t (10) = −3.6; p = .005). There were also differences in MVPA for the Low Active EQ when compared with the control group but the effect was not significant (Mean _diff_ = −5.9 ± 3.0%; t (10) = −2.0; p = .079). Interestingly, the effect of the STEQ treatment in the Low Active was opposite to the effects for ST and EQ with increases in MVPA occurring when compared to the Low Active control group (Mean _diff_ = 6.6 ± 3.0%; t (10) = 2.2; p = .055). Results for Moderate active subgroups in the ST and also in the EQ groups were similar to the results for less active individuals (in the same treatment group). MVPA differences from their respective control group had the same direction but a lower magnitude. Results for the ST and EQ treatments were borderline significant (ST: Mean _diff_ = −7.4 ± 2.6%; t (10) = −2.8; p = .018; EQ: Mean _diff_ = −7.7 ± 2.6%; t (10) = −2.9; p = .015). MVPA difference at trial 2 for individuals in the STEQ treatment had the same direction as their less active peers (exposed to the same treatment) but a lower magnitude. MVPA differences were non-significant (Mean _diff_ =5.5 ± 2.7%; t (10) =2.1; p = .066). These trends reflected some variability around the pooled effect of the Ready for Recess (Figure 3).

**Figure 4:**
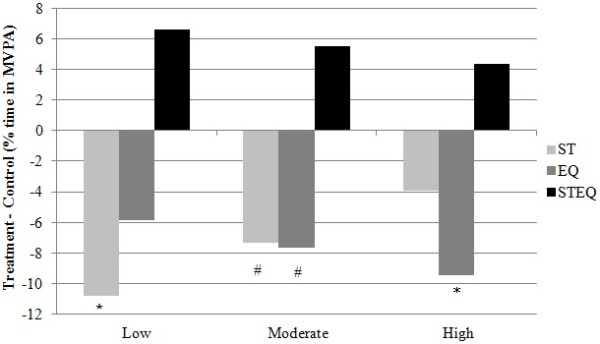
**Average % MVPA difference among Low, Moderate, and High active groups, per treatment group.** Each activity level subgroup was compared with their respective control group (e.g. % MVPA Difference Low Staff = “Low Staff”- “Low Control” at trial 2, holding constant all the remaining variables in the full model). There was a reduced effect trend (from Low to High active individuals) for the Staff (ST), and the Staff + Equipment (STEQ) treatment groups. This trend was reversed in the Equipment (EQ) group (the effect was higher for High active individuals and decreased among Moderate, and Low active subgroups). *Significantly different than respective control group at trial 2 (p < .01). ^#^Borderline significantly different than respective control group at trial 2 (.01 < p < .05).

The most active individuals in the ST (and the STEQ) treatment had the lowest MVPA differences from their respective control group (Staff: Mean _diff_ = −3.9 ± 2.9%; t (10) = −1.3; p = .215; STEQ: Mean _diff_ = 4.4 ± 3.0%; t (10) = 1.5; p = .172). The High Active group in the EQ treatment had the highest MVPA difference from their control group. MVPA levels in this subgroup were significantly lower than the control group (Mean _diff_ = −9.5 ± 2.9%; t (10) = −3.3; p = .009). A summary of the differences between each treatment group and respective control at trial 2 is available in Figure 4.

## Discussion

The present study provides new insights about the outcomes from the R4R project. The main outcome paper from the study [4] reported an overall positive effect of STEQ on boy’s MVPA (+14.1%). Interestingly, there was a decrease on MVPA associated with the ST (boys: -13.5% and girls: -11.4%) and similar results were found for the EQ condition. These results were somewhat surprising since positive effects were expected for both of these independent conditions.

In the present study, we utilized a Latent Growth Curve model to more directly examine the consistency of both individual and subgroup change as a result of the different conditions. In this model, time is defined as a level-1 predictor and the mean (pooled) effects essentially replicate the group patterns evident in the original outcome paper (See Figure 3). Initial analyses demonstrated that there was significant variability around the mean trajectories over time so the nested models focused on predicting individual variation around each of the treatment effects. Baseline levels of MVPA proved to be an important source of variability. These findings were supported as initial MVPA was a significant predictor of MVPA at trial 2 (after 6 months of baseline assessment).

A unique aspect of the present analyses is that we used a similar approach to explore the consistency of this effect for the three primary treatment conditions. The direction of this moderation effect across the three different treatment conditions was specifically examined via higher-order interaction terms (i.e., trial by treatment condition by baseline MVPA). Interestingly, the effect of baseline MVPA on subsequent levels of physical activity was not consistent across the three treatments. The results indicated that initial MVPA moderated the effect of ST, but not EQ and STEQ. In our previous study, we found that ST and EQ were both associated with a pooled negative effect. Participants belonging to schools in these conditions decreased their activity levels after 6 months of intervention [4].

The examination of high-order interactions indicated that change in MVPA levels for children in the ST condition were significantly affected by their baseline MVPA levels. Per each unit increase in baseline MVPA scores, there was a 0.47% attenuation on the differential effect of Staff on MVPA score changes from trial 1 to trial 2. The attenuation means that less active participants were more negatively affected by the presence of trained staff than their more active peers. Surprisingly, the trend was reversed for participants in the EQ condition. The effect was negative but the magnitude of the effect was greater in High Active participants. Nevertheless, the moderation effect of baseline MVPA in this condition was non-significant (p > .05). Less active participants in the STEQ condition reacted more positively to this intervention however, the moderation effect for STEQ was also not significant (possibly due to lack of statistical power).

While the moderation effect was not significant for all treatments, it is noteworthy that the less active children (i.e., those who spent approximately 12% of their recess time in MVPA) were shown to be more responsive to the ST and STEQ conditions. Low active children had declines in time spent in MVPA (~11%) when exposed to staff supervision but increases in MVPA (~6%) when staff utilized recreational equipment and implemented games. Interestingly, similarly to the ST findings, the effect of equipment was also negative but reversed, meaning, those who were more active had greater declines in MVPA. In conclusion, staff training reduced physical activity levels, in low and medium active children.

The examination of these relationships has great value to public health researchers, particularly those interested in promotion strategies for physical activity in elementary children. Many school based interventions have been conducted to promote physical activity behavior in youth. Studies have examined interventions during PE class, in class time, during recess and after school. The common form of analyses conducted in these trials has focused on mean responses of groups of children. The results of the present study provide important insights because it suggests that interventions may have varying effects on different segments of the population. A previous analysis [10] revealed some important differences by gender with regard to physical activity during recess. A recent meta-analysis also suggested that age, and both duration and type of intervention can moderate the effect of PA promotion initiatives during recess [18]. As illustrated by this review, there is a lack of research on the moderation effect of typical activity levels. The present analyses reveal moderation of intervention effects by baseline levels of MVPA. Individuals conducting school-based projects should consider the possible differential effects of programming on different segments of the population. Ideally, interventions should be set up to enhance physical activity opportunities for low active youth [19] without detracting from physical activity opportunities in already active youth.

The results of this study suggest that low active youth are more responsive to changes in the environment of school recess programming, but the effects depend on the nature of the intervention. Staff training sites had staff trained to increase physical activity during recess time. This strategy affected the expected trend in activity from trial 1 to trial 2 (illustrated by the control group) and led to a decrease in activity levels in low active groups. This may be because the structure allowed more active and skilled youth to dominate games. Interestingly, low active individuals’ activity was not negatively affected by the presence of equipment alone. This may be because the equipment gets claimed by more active (and perhaps more dominant) youth in a recess environment. In contrast, activity patterns of low active youth were significantly increased when staff training and equipment were provided (STEQ).

Activity levels of highly active individuals seem to be unaltered when exposed to staff supervision or a combination of both staff and equipment. However, the presence of equipment tended to cause decreases in levels of MVPA. This may be because the equipment became more interesting than the active games that children play when equipment is not provided.

Some limitations of this study should be considered when interpreting the results. As described in the main outcome paper [4], there was no fidelity testing, so it is not possible to quantify the degree of staff involvement and or the compliance with the training provided. This could influence the results and conclusions regarding this condition. Another possible concern is a possible ‘*regression to the mean*’ effect [20] that can be present when baseline measures of the outcome variable are used to predict change over time [21]. Our results indicated that the magnitude of individual MVPA changes over time differed between two conditions (Staff and Equipment) even though group slopes were similar (see Figure 3). Moreover, these changes did not follow similar regression to the mean trends evidenced by the control group. The individual effects were statistically significant for less active individuals in the ST condition but not in the EQ condition. The opposite was found for more active individuals. More active individuals in the EQ condition were significantly affected but similarly active individuals in the ST condition were not significantly affected. Thus, these findings support the idea that differential changes over time in subgroups of individuals could be explained by the R4R intervention and not regression to the mean. Finally, the inclusion of BMI and additional individual-level predictors such as race and/or socio-economical status, in our models could have explained additional variability in the effects of the ready for recess. However these predictors are known to be related with PA and therefore their inclusion might have resulted in over adjustment of our model.

## Conclusions

The findings from this study provide an important contribution to the field of physical activity and public health. Understanding the differential reactivity effect when children are exposed to different PA promotion strategies can help researchers and policy makers choose appropriate intervention designs based on population needs. For example, less active individuals will benefit more from interventions involving supervised staff combined with equipment availability. Children with higher activity levels seem to benefit to a lesser extent. The results from this study could not confirm the broader benefit from this strategy.

This study complements findings from a previous Ready for Recess study [4]. The potential of different strategies to increase active behaviors should be further examined taking into consideration typical activity levels. The detailed and comprehensive examination of the MVPA outcome did not allow a parallel comparison with sedentary behavior. However, this outcome also has important implications for children’s health and therefore should be examined separately.

## Competing interests

The following authors declared no conflicts of interests: PSM, GW, DR, JH. This study was funded by a Robert Wood Johnson Foundation – Active Living Research grant.

## Authors’ contributions

PSM performed the statistical analysis and drafted the manuscript. GW participated in the design of the study and drafted the manuscript. DR participated in the statistical analysis and helped drafted the manuscript. JH conceived of the study, and was responsible for coordination and design. She also helped draft the manuscript. All authors approved the final manuscript.

## Pre-publication history

The pre-publication history for this paper can be accessed here:

http://www.biomedcentral.com/1471-2458/14/103/prepub

## References

[B1] Metcalf B Henley W Wilkin T Effectiveness of intervention on physical activity of children: systematic review and meta-analysis of controlled trials with objectively measured outcomes (EarlyBird 54) BMJ 2012 345 e5888 10.1136/bmj.e5888 23044984

[B2] Centers for Disease Control and Prevention School health guidelines to promote healthy eating and physical activity MMRW, Volume 60 (RR- 5) 2011 1 76 21918496

[B3] Kriemler S Meyer U Martin E van Sluijs EM Andersen LB Martin BW Effect of school-based interventions on physical activity and fitness in children and adolescents: a review of reviews and systematic update Br J Sports Med 2011 45 923 930 10.1136/bjsports-2011-090186 21836176 PMC3841814

[B4] Huberty JL Beets MW Beighle A Saint-Maurice PF Welk G Effects of ready for recess, an environmental intervention, on physical activity in 3rd - 6th grade children J Phys Act Health 2013 in press 10.1123/jpah.2012-0061 23364349

[B5] Ramstetter CL Murray R Garner AS The crucial role of recess in schools J Sch Health 2010 80 517 526 10.1111/j.1746-1561.2010.00537.x 21039550

[B6] Pellegrini A Smith P School recess: implications for education and development Review of Educational Research 1993 63 51 67 10.3102/00346543063001051

[B7] Erwin H Abel M Beighle A Noland MP Worley B Riggs R The contribution of recess to children's school-day physical activity J Phys Act Health 2012 9 442 448 21934153 10.1123/jpah.9.3.442

[B8] Ridgers ND Saint-Maurice PF Welk GJ Siahpush M Huberty J Differences in physical activity during school recess J Sch Health 2011 81 545 551 10.1111/j.1746-1561.2011.00625.x 21831067

[B9] Ridgers ND Timperio A Crawford D Salmon J Five-year changes in school recess and lunchtime and the contribution to children's daily physical activity Br J Sports Med 2012 46 741 746 10.1136/bjsm.2011.084921 21596716

[B10] Saint-Maurice PF Welk GJ Silva P Siahpush M Huberty J Assessing children's physical activity behaviors at recess: a multi-method approach Pediatr Exerc Sci 2011 23 585 599 22109782 10.1123/pes.23.4.585

[B11] Ridgers ND Salmon J Parrish AM Stanley RM Okely AD Physical activity during school recess: a systematic review Am J Prev Med 2012 43 320 328 10.1016/j.amepre.2012.05.019 22898126

[B12] Connolly P TL M Effects of a game intervention on the physical activity levels of children at recess Res Q Exerc Sport 1995 66 A66

[B13] Verstraete SJ Cardon GM De Clercq DL De Bourdeaudhuij IM Increasing children's physical activity levels during recess periods in elementary schools: the effects of providing game equipment Eur J Public Health 2006 16 415 419 10.1093/eurpub/ckl008 16431866

[B14] Parrish AM Okely AD Stanley RM Ridgers ND The effect of school recess interventions on physical activity: a systematic review Sports Med 2013 43 287 299 10.1007/s40279-013-0024-2 23512170

[B15] Bryk SA Raudenbush SW Hierarchical Linear Models - Applications and Data Analysis Methods 1992 2 Thousand Oaks, CA: Sage Publications

[B16] Preacher KJ Wichman AL MacCallum RC Briggs NE Latent Growth Curve Modeling 2008 Thousands Oaks, CA: Sage Publications

[B17] Freedson P Pober D Janz KF Calibration of accelerometer output for children Med Sci Sports Exerc 2005 37 S523 S530 10.1249/01.mss.0000185658.28284.ba 16294115

[B18] Erwin HE Ickes M Ahn S Fedewa A Impact of recess interventions on children's physical activity-a meta-analysis Am J Health Promot 2014 28 159 167 10.4278/ajhp.120926-LIT-470 23875990

[B19] Fairclough SJ Beighle A Erwin H Ridgers ND School day segmented physical activity patterns of high and low active children BMC Public Health 2012 12 406 10.1186/1471-2458-12-406 22672654 PMC3434066

[B20] Bland JM Altman DG Regression towards the mean BMJ 1994 308 1499 10.1136/bmj.308.6942.1499 8019287 PMC2540330

[B21] Vickers AJ Altman DG Statistics notes: analysing controlled trials with baseline and follow up measurements BMJ 2001 323 1123 1124 10.1136/bmj.323.7321.1123 11701584 PMC1121605

